# Characterization of *TCF3* rearrangements in pediatric B-lymphoblastic leukemia/lymphoma by mate-pair sequencing (MPseq) identifies complex genomic rearrangements and a novel *TCF3/TEF* gene fusion

**DOI:** 10.1038/s41408-019-0239-z

**Published:** 2019-10-01

**Authors:** Ross A. Rowsey, Stephanie A. Smoley, Cynthia M. Williamson, George Vasmatzis, James B. Smadbeck, Yi Ning, Patricia T. Greipp, Nicole L. Hoppman, Linda B. Baughn, Rhett P. Ketterling, Jess F. Peterson

**Affiliations:** 1 Division of Laboratory Genetics and Genomics, Department of Laboratory Medicine and Pathology, Mayo Clinic, Rochester, MN USA; 2 Center for Individualized Medicine-Biomarker Discovery, Mayo Clinic, Mayo Clinic, Rochester, MN USA; 3 Department of Pathology, Johns Hopkins University School of Medicine, Baltimore, MD USA

**Keywords:** Cytogenetics, Genetic translocation

## Abstract

The *TCF3/PBX1* gene fusion is a recurrent genetic abnormality in pediatric B-lymphoblastic leukemia/lymphoma (B-ALL/LBL). While dual-color, dual-fusion fluorescence in situ hybridization (D-FISH) probes can detect *TCF3/PBX1* fusions, further characterization of atypical *TCF3* FISH patterns as indicated by additional or diminished *TCF3* signals is currently limited. Herein we describe the use of a next-generation sequencing assay, mate-pair sequencing (MPseq), to characterize typical and cryptic *TCF3/PBX1* fusions and to identify *TCF3* translocation partners based on results obtained from our laboratory-developed *TCF3/PBX1* D-FISH probe set. MPseq was performed on 21 cases of pediatric B-ALL/LBL with either *TCF3/PBX1* fusion, or no *TCF3/PBX1* fusion but with additional or diminished *TCF3* signals obtained by our *PBX1/TCF3* D-FISH probe set. In addition, MPseq was performed on one pediatric B-ALL/LBL case with an apparently normal karyotype and abnormal *TCF3* break-apart probe results. Of 22 specimens successfully evaluated by MPseq, 13 cases (59%) demonstrated *TCF3/PBX1* fusion, including three cases with previously undescribed insertional rearrangements. The remaining nine cases (41%) harbored various *TCF3* partners, including six cases with *TCF3/ZNF384*, and one case each with *TCF3/HLF*, *TCF3/FLI1* and *TCF3/TEF*. Our results illustrate the power of MPseq to characterize *TCF3* rearrangements with increased precision and accuracy over traditional cytogenetic methodologies.

## Introduction

Rearrangements involving the *TCF3* (previously known as *E2A*) gene region (19p13.3) are common in both pediatric and adult B-lymphoblastic leukemia/lymphoma (B-ALL/LBL) and account for ~6% of newly diagnosed cases^1–4^. The *PBX1* gene (1q23) is the most common translocation partner for TCF3, resulting in *TCF3/PBX1* gene fusion, and is currently classified in the WHO as a recurrent genetic abnormality in B-ALL/LBL^4^. Fusion of *TCF3/PBX1* is usually generated from a reciprocal t(1;19)(q23;p13.3) and results in a 5′*TCF3/*3′*PBX1* fusion gene located on the der(19)t(1;19) chromosome^4–6^. Interestingly, the majority of 1;19 translocations (~80%) only present with the der(19)t(1;19) as observed by conventional chromosome and/or fluorescence in situ hybridization (FISH) studies^5^. Several rare *TCF3* gene fusion partners have been described, most commonly *ZNF384* (12p13) and *HLF* (17q21)^7–9^. Since highly variable prognoses are associated with the various *TCF3* translocation partners (*TCF3/HLF* fusion has an extremely poor prognosis in contrast to the favorable/standard risk for *TCF3/PBX1* fusion), the characterization of *TCF3* partners is essential^4^.

To detect the recurrent *TCF3/PBX1* gene fusion in B-ALL/LBL, our laboratory developed and validated a dual-color, dual-fusion FISH (D-FISH) *TCF3/PBX1* probe set for clinical application^10^. To further characterize *TCF3/PBX1* fusions associated with discordant chromosome results and the *TCF3* rearrangements with additional or diminished *TCF3* signals obtained by our *TCF3/PBX1* D-FISH probe set, we utilized a next-generation sequencing (NGS) strategy, mate-pair sequencing (MPseq). This novel NGS-based technology enables the characterization of chromosomal rearrangements with significantly higher resolution and precision compared to conventional cytogenetic methodologies, including chromosome and FISH studies. Herein, we report conventional chromosome, FISH and the molecular characterization of each *TCF3* rearrangement by MPseq from 22 patients with pediatric B-ALL/LBL. This study provides a molecular window into the complexity resulting in *TCF3/PBX1* fusion and also highlights the importance of characterizing variant *TCF3* partner genes by NGS methods such as MPseq.

## Materials and methods

### Patient selection

Following institutional review board approval, a retrospective review of the Mayo Clinic cytogenetic database was performed to identify bone marrow samples with abnormal results evaluated by our *TCF3/PBX1* D-FISH probe set. Ten pediatric patient cases were chosen that had typical concordant abnormal chromosome and abnormal *TCF3/PBX1* fusion D-FISH signal patterns, including six patients with a balanced t(1;19) and a 1R1G2F D-FISH signal pattern (Table 1; patients 1–6), and four patients with an unbalanced der(19)t(1;19) and a 2R1G1F D-FISH signal pattern (Table 1; patients 7–10). In addition, three pediatric patient cases were included with discordant abnormal chromosome results versus D-FISH results (Table 1; patients 11–13). Patients with atypical D-FISH signal patterns including additional or diminished *TCF3* signals in the absence of *TCF3/PBX1* fusion were also identified, and represent a total of eight cases (Table 1; patients 14–21). One case with an apparently normal female karyotype and *TCF3* rearrangement obtained via a *TCF3* break-apart probe (BAP) strategy was also included in our study (Table 1; patient 22). In total, 22 cases of pediatric B-ALL/LBL with *TCF3* abnormalities detected by FISH were included in our study, and evaluated by MPseq on either fresh or fixed cell pellets from bone marrow aspirate specimens.

**Table 1:** Conventional cytogenetic, *PBX1/TCF3* D-FISH and MPseq results for 22 cases of pediatric B-ALL with *TCF3* rearrangements

Patient	Gender	Age (years)	Karyotype	Interphase *PBX1/TCF3* D-FISH signal patterns	Abnormal interphase nuclei (%)	MPseq results	*TCF3* fusion partner	Exon fusion (*TCF3*; partner)
1	F	2	46,XX,t(1;19)(q23;p13.3)[1]/46,XX,idem,psu dic(9;6)(p13;q13)[15]/46,XX[4]	(PBX1,TCF3)x3(PBX1 con TCF3x2)	91	t(1;19)	*PBX1*	e16;e3
2	M	2	46,XY,t(1;19)(q23;p13.3)[11]/46,XY[9]	(PBX1,TCF3)x3(PBX1 con TCF3x2)	86	t(1;19)	*PBX1*	e16;e3
3	M	5	46,XY,t(1;19)(q23;p13.3)[8]/49,idem,+6,+21,+22[8]/46,XY[4]	(PBX1,TCF3)x3(PBX1 con TCF3x2)	95	t(1;19)	*PBX1*	e16;e3
4	M	7	46,XY,t(1;19)(q23;p13.3)[17]/46,XY[3]	(PBX1,TCF3)x3(PBX1 con TCF3x2)	98	t(1;19)	*PBX1*	e16;e3
5	M	16	46,XY,t(1;19)(q23;p13.3)[16]/46,XY[4]	(PBX1,TCF3)x3(PBX1 con TCF3x2)	92	t(1;19)	*PBX1*	e16;e3
6	M	16	46,XY,t(1;19)(q23;p13.3)[15]/46,XY[5]	(PBX1x3~4,TCF3x3(PBX1 con TCF3x2)	96	t(1;19)	*PBX1*	e16;e3
7	F	4	46,XX,add(8)(q22),add(9)(p13),del(9)(p13p22),der(19)t(1;19)(q23;p13.3)[20]	(PBX1x3,TCF3x2)(PBX1 con TCF3x1)	95	der(19)t(1;19)	*PBX1*	e16;e3
8	M	4	46,XY,der(19)t(1;19)(q23;p13.3)[2]/46,idem,−6,del(13)(q12q22),+mar[4]/46,XY[14]	(PBX1x3,TCF3x2)(PBX1 con TCF3x1)	98	der(19)t(1;19)	*PBX1*	e16;e3
9	F	9	46,XX,+1,der(1;22)(q10;q10),der(19)t(1;19)(q23;p13.3)[5]/46,idem,del(13)(q12q22)[8]/46,XX[7]	(PBX1x4,TCF3x2)(PBX1 con TCF3x1)	97	der(19)t(1;19)	*PBX1*	e16;e3
10	F	9	46,XX,del(6)(q13q23),der(19)t(1;19)(q23;p13.3)[9]/46,XX[11]	(PBX1x3,TCF3x2)(PBX1 con TCF3x1)	96	der(19)t(1;19)	*PBX1*	e16;e3
11	M	4	46,XY,der(19)t(1;19)(q23;p13.3)[13]/46,XY[7]	(PBX1,TCF3)x3(PBX1 con TCF3x2)	83	Complex	*PBX1*	e16;e3
12	M	8	46,XY,der(19)t(1;19)(q23;p13.3)[2]/46,XY[18]	(PBX1,TCF3)x3(PBX1 con TCF3x2)	72	Complex	*PBX1*	e16;e3
13	M	15	46,XY,der(19)t(1;19)(q23;p13.3)[10]/46,XY[10]	(PBX1,TCF3)x3(PBX1 con TCF3x2)	69	Complex	*PBX1*	e17;e5
14	F	2	46,XX,t(16;16)(p13.3;q22),add(19)(p13.2)[16]/46,XX[4]	(PBX1x2,TCF3x3)	56	t(12;19)	*ZNF384*	e13;e3
15	M	6	NA	(PBX1x2,TCF3x3)	62	t(12;19)	*ZNF384*	e17;e7
16	F	8	NA	(PBX1x2,TCF3x3)	47	t(12;19)	*ZNF384*	e13;e3
17	F	13	48,X,add(X)(q13),+2,der(5)add(5)(p12)t(5;10)(q31;q22),+8,idic(9)(p10),der(10)t(5;10)(q31;q22),t(12;14)(q11;p11.2),add(15)(q22), add(19)(p13.3),+mar[18]/46,XX[2]	(PBX1x2,TCF3x3)	33	t(12;19)	*ZNF384*	e13;e3
18	F	1	46,XX,add(19)(p13.3)[8]/46,idem,add(5)(q13),del(14)(q13)[1]/46,XX[11]	(PBX1x2,TCF3 dimx1,TCF3x1)	76	der(19)t(12;19)	*ZNF384*	e11;e3
19	M	8	46,XY,der(3)(3pter->3q13.2::3q26.3->3qter),der(9)(9pter->9p22::3q13.2->3q26.2::9p13->9qter)[5]/46,XY[15]	(PBX1x2,TCF3 dimx1,TCF3x1)	77	der(19)t(12;19)	*ZNF384*	e13;e2
20	F	3	46,XX,add(9)(p22)[2]/46,XX,t(2;9)(p13;p22)[2]/46,XX[16]	(PBX1x2,TCF3x3)	31	t(19;22)	*TEF*	e16;e4
21	F	10	46,XX,der(11)t(11;19)(q23;p13.1),add(12)(p11.2)[2]/46,XX[18]	(PBX1x2,TCF3x3)	79	t(11;19)	*FLI1*	e16;e6
22^a^	F	13	46,XX[20]	(5′TCF3,3′TCF3)x2(5′TCF3 sep 3′TCF3x1)	69	t(17;19)	*HLF*	?e14–16;e4

*FISH* fluorescence in situ hybridization, *D-FISH* dual-color dual-fusion FISH probe set, *MPseq* mate-pair sequencing

^a^Karyotype and *TCF3* break-apart FISH probe results reported by an outside institution

### Conventional chromosome analysis

Bone marrow aspirate specimens were cultured in unstimulated media, harvested and banded utilizing standard cytogenetic techniques according to specimen-specific protocols^11^. Twenty metaphases were analyzed when available.

### Fluorescence in situ hybridization

FISH analysis was performed on bone marrow aspirates specimens following standard FISH pretreatment, hybridization and fluorescence microscopy protocols utilizing a laboratory-developed *TCF3/PBX1* D-FISH probe set. Details regarding probe development and performance have been previously described^10^.

### Mate-pair sequencing

MPseq was performed using the Illumina Nextera Mate-Pair library protocol (Illumina, San Diego, CA) and sequenced on the Illumina HiSeq 2500. Sequence data were aligned to hg38 using BIMA, and a custom bioinformatics tool was used to filter the alignments and identify junctions. Detailed methods can be found in Drucker et al., Johnson et al., and Smadbeck et al.^12–14^.

## Results

Conventional chromosome, FISH, and MPseq results from all 22 patients (age range: 1–16 years) are presented in Table 1. For the 13 patients with *TCF3/PBX1* fusion, six cases had a balanced t(1;19) by chromosomes and a double fusion by D-FISH (patients 1–6), four cases had an unbalanced der(19)t(1;19) by chromosomes and a single fusion by D-FISH (patients 7–10), and three cases had seemingly discrepant chromosome and FISH results, indicated by an unbalanced der(19)t(1;19) by chromosomes versus a double fusion signal pattern by D-FISH (patients 11–13).

**Fig. 1:**
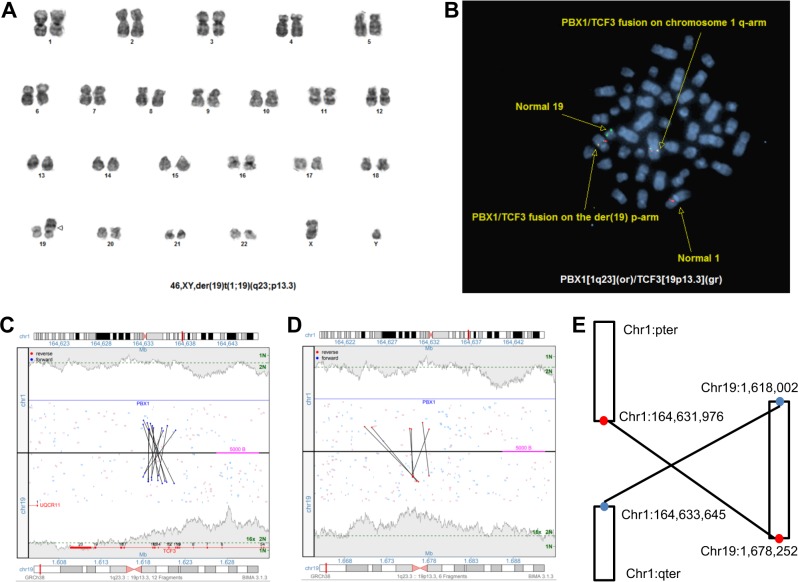
Genomic studies from patient 11, including **a** representative karyogram with a der(19)t(1;19). **b** Metaphase FISH illustrating one *TCF3/PBX1* fusion on the der(19)t(1;19) and one *TCF3/PBX1* fusion on a “normal” chromosome 1q. **c** MPseq junction plots demonstrating the fusion-generating translocation, and **d** the secondary junction demonstrating insertion. **e** Graphic demonstrating the insertional mechanism represented by the junction plots, with ~60 kb of chromosome 19 material inserted into chromosome 1. Breakpoints represent approximate breakpoint estimated by the MPseq pipeline

MPseq confirmed the balanced (patients 1–6) and unbalanced (patients 7–10) 1;19 translocations observed by both chromosome and FISH studies. In addition, MPseq revealed insertional rearrangements accounting for the discrepant chromosome and FISH results in the three cases with der(19)t(1;19) and two fusion signals (patients 11–13) (Fig. 1a–e). The MPseq results explained serial metaphase FISH, which had documented *TCF3/PBX1* fusion located on a “normal” copy of chromosome 1q in each case, suggesting a cryptic insertional rearrangement.

**Fig. 2:**
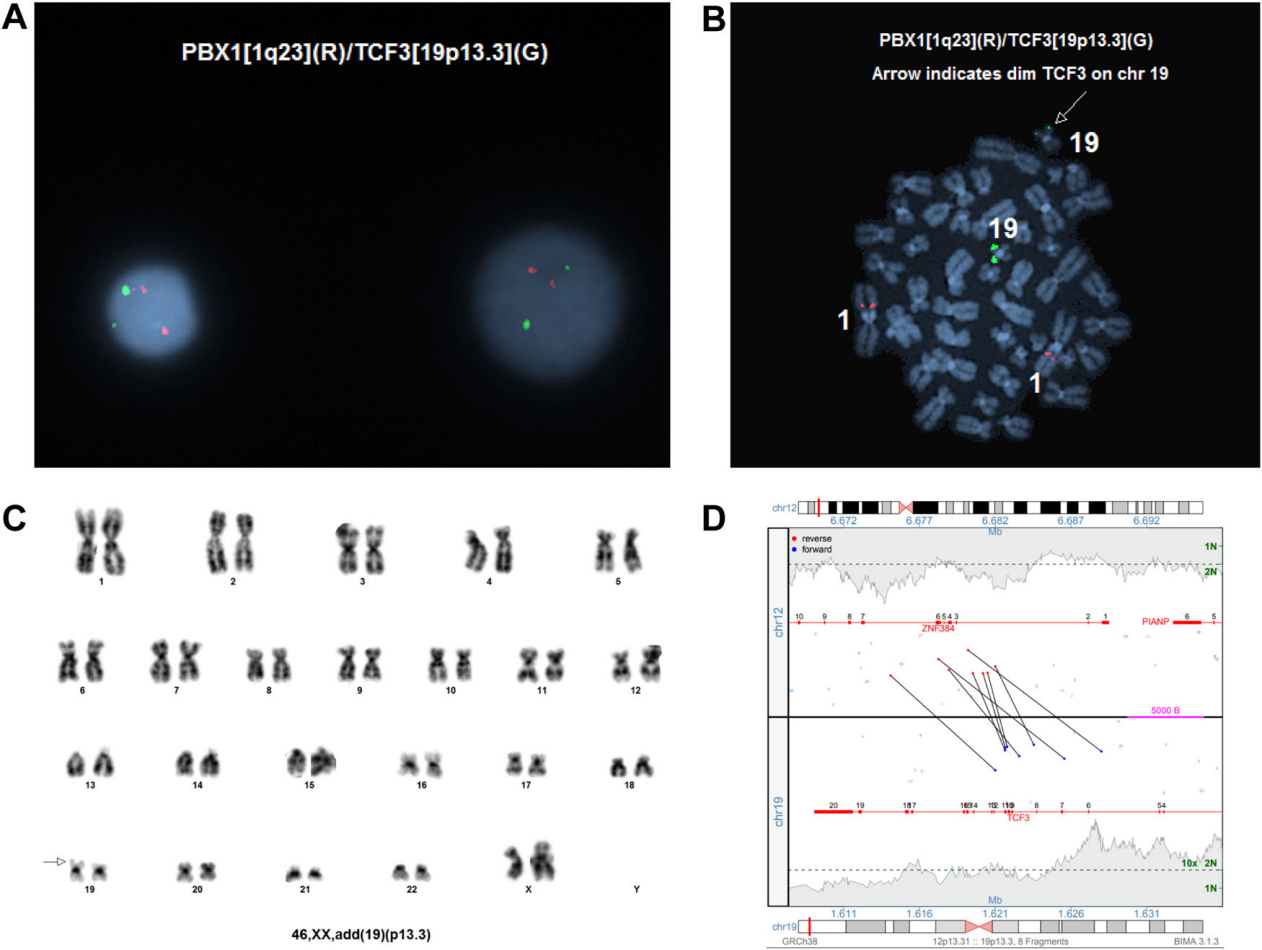
Genomic studies from patient 18, including **a** representative interphase cells showing a diminished *TCF3* signal suggesting a partial deletion or rearrangement of *TCF3*, using the *TCF3/PBX1* D-FISH probe set. **b** Metaphase FISH demonstrating the diminished *TCF3* signal is retained on chromosome 19p. **c** Representative karyogram with chromatin of undetermined origin attached to 19p. **d** MPseq junction plot demonstrating a *TCF3/ZNF384* fusion

**Fig. 3:**
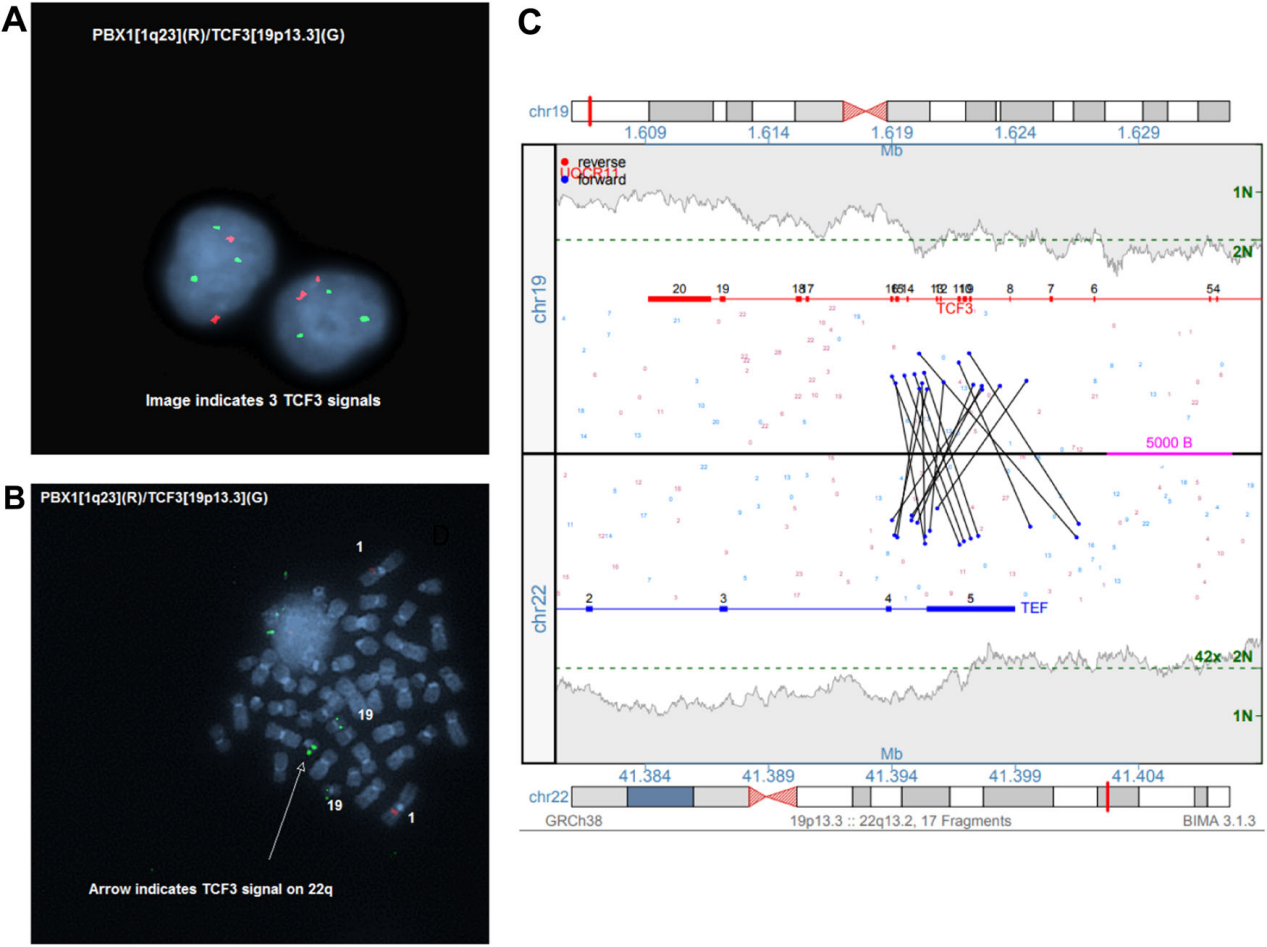
Genomic studies from patient 20, including **a** interphase cells showing an extra *TCF3* signal using the *TCF3/PBX1* D-FISH probe set. **b** Metaphase FISH showing hybridization of *TCF3* signals to both chromosomes 19, in addition to 22q. **c** MPseq junction plot demonstrating *TCF3/TEF* fusion

The nine remaining samples (patients 14–22) had additional or diminished *TCF3* signals in the absence of *TCF3/PBX1* fusions, and only four of seven cases (patients 14, 17, 18 and 21) with available chromosome studies had abnormalities involving the 19p13 chromosomal region. Among these nine cases, MPseq detected variant *TCF3* gene partners, including six cases with *ZNF384* (patients 14–19) (Fig. 2a–d), and one case each with the following three genes: *TEF* (patient 20) (Fig. 3a–c), *FLI1* (patient 21) and *HLF* (patient 22). Moreover, MPseq indicated all rearrangements were predicted to create in-frame gene–gene fusions, including all three potential *TCF3* exons (14–16) described in patient 22.

## Discussion

Herein, we demonstrate the ability for MPseq to molecularly characterize 22 B-ALL/LBL specimens that were either positive for *TCF3/PBX1* fusion or displayed an atypical *TCF3* FISH pattern with additional or diminished *TCF3* signals obtained by our *TCF3/PBX1* D-FISH probe set. Precise identification of these rearrangements is critical as there are significant prognostic differences between *TCF3/PBX1* fusion and the less common *TCF3/HLF* fusion, the latter having a significantly unfavorable prognosis^4,9,15^.

To our knowledge, this represents only the second report of *TCF3/FLI1* fusion in the literature and the first to report *TCF3/TEF* fusion^16^. *TCF3* is a helix–loop–helix (HLH) transcription factor critical for lymphopoiesis in B- and T-cell lineage development^17,18^. When the transactivation domain of *TCF3* (N-terminal region) is separated from its intrinsic DNA-binding domain (C-terminal) and forms a chimeric fusion with a DNA-binding domain from a new gene partner, the subsequent chimeric fusion protein acts as an oncogenic driver^17,18^. Functional similarities have been described between the DNA binding domains of the *TEF* and *HLF* genes, the latter being the *TCF3* gene fusion partner observed in the prognostically unfavorable t(17;19)^19,20^. Since these two *TCF3* translocation partners share distinct functional similarities, this suggests that the t(19;22) likely represents the oncogenic fusion and similarly implicates *TEF* as another potentially aggressive partner of *TCF3*, although additional follow-up for our patient and identification of the 19;22 translocation and outcomes in other patients are necessary.

Ten pediatric B-ALL/LBL cases with both balanced (patients 1–6) and unbalanced (patients 7–10) *TCF3/PBX1* fusions, as initially identified by conventional chromosome and *TCF3/PBX1* D-FISH studies, were readily confirmed by MPseq. In addition, three *TCF3/PBX1* fusion cases (patients 11, 12 and 13) with discrepant chromosome [der(19)t(1;19)] and D-FISH results (two fusions) were further characterized by MPseq beyond the capability of traditional chromosome and FISH methodologies. In all three cases, D-FISH studies suggested a typical 1;19 translocation (two fusion signal pattern); however, the conventional chromosome study in each case identified the more common unbalanced derivative chromosome 1;19 along with two apparently normal copies of chromosome 1. Metaphase FISH studies using the *TCF3/PBX1* probe set on the abnormal metaphases demonstrated the expected *TCF3/PBX1* fusion on the der(19)t(1;19), while the second *TCF3/PBX1* fusion was revealed on one of the apparently normal copies of chromosome 1 at the typical 1q23 location of *PBX1*, suggesting a potential cryptic insertional rearrangement. MPseq clarified that an ~30–60 kb segment of chromosome 19p was inserted into chromosome 1q, supporting the D-FISH results and resulting in a complex insertion involving two chromosomal regions containing the *TCF3/PBX1* fusion. This insertional translocation and the derivative chromosome 19 show only a single breakpoint resulting in *TCF3/PBX1* fusion, likely indicating an initial translocation event followed by a secondary “repair” event to restore the karyotypically normal chromosome 1. Alternatively, and less likely, insertional and translocation events with nearly identical breakpoints occurred independently, each resulting in a *TCF3/PBX1* fusion.

MPseq also revealed two cryptic *TCF3/ZNF384* fusions resulting from unbalanced chromosome 19p translocation events (patients 18 and 19). These cases resulted in ~6 Mb of chromosome 12 material replacing ~2 Mb of chromosome 19 material, a subtle change that is not readily detectable by conventional chromosome analysis. Additionally, since this rearrangement results in the loss of only a portion of the *TCF3* probe signal using our t(1;19) D-FISH strategy, the only visible alteration was a diminished *TCF3* signal.

In cases with additional *TCF3* signals identified by interphase *TCF3/PBX1* D-FISH studies, metaphase FISH may provide low-resolution input into the general chromosomal locus of the *TCF3* gene partner. For patients 14, 20 and 21, metaphase FISH localized the additional *TCF3* FISH signal to chromosomes 12p, 22q and 11q, respectively. However, for patients 15, 16 and 17, metaphase FISH analysis was not possible due to either absence of metaphases or poor sample quality. Irrespective of metaphase FISH studies, which can at best suggest potential partner genes, MPseq was necessary to characterize each of the *TCF3* gene fusion partners in nine cases evaluated in our series, including *ZNF384* (patients 14–19), *TEF* (patient 20), *FLI1* (patient 21) and HLF (patient 22).

MPseq therefore represents an advance in the detection of clinically relevant structural rearrangements^21–23^. While traditional NGS has the potential to detect rearrangements using paired end sequencing, MPseq allows for the characterization of structural rearrangements while requiring a significantly lower depth of coverage. The strength of MPseq lies in the unique library preparation, where long reads (2–5 kb) are circularized and fragmented to allow for their interrogation using traditional NGS. In contrast to basic NGS, where paired end sequencing will give linked reads separated by a few hundred base pairs, the circular MPseq fragments give linked reads much further away from each other (2–5 kb), thus significantly increasing the potential to identify discordant reads^21^.

The ability to assess genome-wide structure down to gene-level resolution makes MPseq a powerful tool for annotating simple and complex structural rearrangements as well as copy number changes, although some limitations exist. Breakpoints occurring within repetitive regions, such as in centromeric or segmental duplication mediated regions are challenging to map. The ability to use overall coverage as a proxy for copy number alleviates this limitation to some extent assuming any repeat-mediated rearrangement is unbalanced. Similarly, its ability to detect terminal rearrangements is also limited since telomeric sequences are also repetitive and difficult to map, and are therefore subject to the same technical challenges. Lastly, the low depth of coverage of MPseq makes the detection of low level or subclonal rearrangements more challenging, with clonal populations at less than 25 and 10% involvement having reduced sensitivity for copy number and structural rearrangements, respectively^23^. Therefore, MPseq offers the most utility in a diagnostic setting as opposed to monitoring for minimal residual disease. However, as the cost of sequencing continues to decline and instrument output increases, it is likely these limitations could be overcome with increased sequencing depth.

In conclusion, we have presented a 22-patient pediatric B-ALL/LBL cohort with *TCF3* rearrangements that have been interrogated by standard cytogenetics, D-FISH for *TCF3/PBX1* and MPseq. Our data demonstrate the ability of MPseq to characterize cryptic structural rearrangements associated with discordant chromosome and *TCF3/PBX1* FISH results and to identify rare and novel *TCF3* gene fusion partners associated with diminished or additional *TCF3* FISH signals. We report the identification of a novel *TCF3* fusion partner, *TEF*, by MPseq, and based on the functional similarities to *HLF*, *TEF* may represent a second possible unfavorable *TCF3* translocation. Overall, MPseq represents a powerful NGS-based technology that allows for further investigation and characterization of structural chromosome rearrangements in hematologic malignancies and will aid in the overall understanding of genomic structural variation.
